# Pre-Treatment and Post-Treatment Dental Anxiety in Patients Visiting Intern Dental Clinic

**DOI:** 10.3390/medicina59071284

**Published:** 2023-07-11

**Authors:** Abdulaziz Alsakr, Khalid Gufran, Abdullah Saad Alqahtani, Hassan Alkharaan, Alwaleed Abushanan, Banna Alnufaiy, Abdullah Alkhaldi, Tareq Alshammari, Muhannad Alanazi

**Affiliations:** 1 Department of Preventive Dental Sciences, College of Dentistry, Prince Sattam Bin Abdulaziz University, Alkharj 11942, Saudi Arabia; k.syed@psau.edu.sa (K.G.); ab.alkahtani@psau.edu.sa (A.S.A.); h.alkharaan@psau.edu.sa (H.A.); a.abushanan@psau.edu.sa (A.A.); b.alnoufaiy@psau.edu.sa (B.A.); 2 College of Dentistry, Prince Sattam Bin Abdulaziz University, Alkharj 11942, Saudi Arabia; alkhaldi12263@gmail.com (A.A.); dr.tareq28@gmail.com (T.A.); al.onaizi39@gmail.com (M.A.)

**Keywords:** dental anxiety, MDAS, intern dental doctor

## Abstract

*Background and Objectives*: This study aimed to assess the dental anxiety of patients using the modified dental anxiety scale (MDAS) questionnaire along with examining the possible relationship between dental anxiety and sociodemographic factors. *Materials and Methods*: The MDAS questionnaire was used to assess the anxiety level of the patients which included a total of five questions and five options to respond to each question. MDAS questionnaire was filled out by all the patients before the dental treatment. After finishing the treatment, all the patients were given a post-treatment questionnaire to assess their anxiety levels after treatment. Descriptive statistics were performed for measuring the frequency of responses. Mann-Whitney U test was used to compare the anxiety between the gender. The chi-square test was used to identify the distribution of pre-treatment with gender and post-treatment questionnaire with gender and treatment modalities. Linear regression was used to identify the association between clinical variables and different levels of anxiety. *Results*: A total of 115 patients including 59 males and 56 females with a mean age of 35 ± 11.34 years were included in the current study. Female patients were significantly more anxious about dental treatment compared to male patients. Linear regression showed that age and gender have a significant association with the pre-treatment anxiety level; however, types of treatment is not associated with the MDAS. Anxiety levels decreased for the majority of the patients after the treatment and types of treatment did not show any differences with the post-treatment anxiety level. *Conclusions*: Age and gender play an important role in dental anxiety; however, types of treatment are not associated with pre-treatment and post-treatment dental anxiety.

## 1. Introduction

Intense anxiety or fear that an individual feel in the dental setting is referring as dental anxiety. This is a quite common incident; therefore, it is a concern of public health as dental anxiety unfavorably affects the oral health of dental patients [1]. Moreover, the patients with dental anxiety required to refer to the dental professionals which causes higher costs for national health systems. In addition, it generally takes more time to treat patients with anxiety which steal time from other potential treatments on same patient or on other patients. The occurrence of dental anxiety ranges from four to 20% in developed countries including some variations due to the different usage of dental anxiety scales [2]. Individuals who are suffering from dental anxiety tend to have untreated carious teeth, missing teeth, and poor oral health [1,3,4,5]. Moreover, people with dental anxiety specifically younger individuals and females seem to avoid dental appointments which leads to poorer dental health due to the delay of scheduled treatment [1,5,6,7,8,9].

As dental anxiety is a common phenomenon, this should be diagnosed properly before attending a dentist using a reliable and valid diagnostic tool. Therefore, the researcher established different modalities to ascertain dental anxiety. The modified dental anxiety scale (MDAS) is one of the most used questionnaires to assess dental anxiety among patients attending a dentist irrespective of the dental procedures [10,11,12]. MDAS was adapted from the original Corah’s dental anxiety scale (CDAS) which consists of four questions to evaluate the anxiety level of the individual. Later on, CDAS was modified by Humphris et al. [12] and added an additional question about local anesthesia and the responses from ‘not anxious’ to ‘extremely anxious’. This questionnaire was established in the English language and used in many English-speaking countries [13,14,15]. Due to the popularity of MDAS, this questionnaire was translated and validated in other languages such as Japanese [16], Spanish [17], French [8], Malay [18], Chinese [19], Tamil [20], Italian [21], Turkish [22], Greek [23], Nepali [24] and Arabic [25,26,27,28,29].

It mentioned in the previous study that dental anxiety could have increased among the patients if there was any unpleasant previous experience persisted [30]. The dental procedures which required anesthesia and rotative instruments like direct restorations [31] or indirect ones [32] creates more anxiousness among the patients. Patients who have dental anxiety report more pain in some dental procedures such as deep fillings, root canal therapy, subgingival scaling, and extractions [33]. The negative and painful dental experiences could be responded due to the efficiency of the treatment and the personal quality of the dentists [30]. Pain related with the dental treatment might have influence in terms of the cooperation of the dental treatment. These painful experience in the dental clinic even carry out in the adulthood [34]. Therefore, previous study worked on the computer assisted anesthesia which showed significantly low scores in pain perception compared to the traditional injectable anesthesia and yield cooperative behavior [35].

A dental hospital usually comprises different ranks of dental practitioners including specialists, registrars, general dentists, intern dental doctors, and dental students. Though the quality of dental treatment differs regardless of the professional rank, the satisfaction level of the patients might vary depending on by whom the treatment was provided. There were studies that assessed the dental anxiety of the patients who visited dental clinics or hospitals [25,27,36,37,38,39]. However, few studies have been conducted on the dental anxiety of patients who attended student dental clinics [40,41] and intern dental doctors [42,43]. Assessing the dental anxiety level who are treated by students or intern doctors is important as anxiety level differs with the rank of practitioners [41,43]. As per the literature, no study was conducted on patients’ dental anxiety treated by intern doctors using the MDAS questionnaire. Therefore, this study aimed to assess the dental anxiety of patients using the MDAS questionnaire along with examining the possible relationship between dental anxiety and sociodemographic factors.

## 2. Materials and Methods

This current prospective study was conducted at the College of Dentistry, Prince Sattam Bin Abdulaziz University. The standing committee of bioethics research (SCBR) of Prince Sattam Bin Abdulaziz University approved this study protocol (SCBR-079-2022). Moreover, the study was conducted according to the guidelines of the Declaration of Helsinki.

Patients who were seeking dental treatment in the intern dental clinic of Prince Sattam Bin Abdulaziz University were included in this study after carefully assessing the inclusion and exclusion criteria of this study (Table 1).

The dental intern doctors of Prince Sattam Bin Abdulaziz University are treating patients who are new and visit the clinics for receiving routine dental care. The intern doctors were specifically chosen for this study as they attend to a higher proportion of new patients and perform comprehensive dentistry. All the patients were conveniently selected until the data collection period for this research.

A paper version of MDAS questionnaire [40] was provided to all the patients before starting the treatment to assess the anxiety level of the patients. MDAS questionnaire consists of five questions and five options (not anxious, slightly anxious, fairly anxious, very anxious, and extremely anxious) for each question to be answered by the patients. The questionnaire is attached as a Supplementary Document. The responses were scored from one to five based on the severity of anxiousness. ‘Not anxious’ scored one and ‘extremely anxious’ scored five. All the scores were added across all the questions and the highest possible score was 25. A cut-off score was set at 19 to determine high-level anxiousness and low-level anxiousness. MADS score ≥ 19 is considered high-level anxiousness and <19 is considered low-level anxiousness [40]. Other clinical information such as age, gender, ethnicity, and required treatment was also recorded along with the questionnaire from each patient.

In order to assess the impact of treatment by intern doctors and the anxiety level of the patients after the treatment, another questionnaire was given to all the patients after finishing the treatment which consists of five questions similar to the MDAS questionnaire [36]. The post-treatment questionnaire is attached as a Supplementary Document. All the treatments were carried out by the dental intern doctors under the supervision of specialists.

All the statistical analyses were performed using the IBM SPSS version 27 (IBM, Armonk, NY, USA). Descriptive data were analyzed with frequency distribution. A normality test was performed to assess the normality of the data. Pre-treatment dental anxiety between the gender was compared with the Mann-Whitney U test. The chi-square test was used to identify the distribution of pre-treatment with gender and post-treatment questionnaire with gender and treatment modalities. Linear regression was performed to identify the association of sociodemographic factors with dental anxiety.

## 3. Results

A total of 115 patients including 59 males and 56 females with a mean age of 35 ± 11.34 years were included in the current study. Kolmogorov-Smirnov statistics was used to assess the normality of the data and it presented that data were not normally distributed. Therefore, non-parametric data was used. Descriptive statistics of the participants are exhibited in Table 2. Pre-treatment was given to all patients before starting the treatment. The frequency of their responses showed in Table 3. It showed that the majority of the patients (34.09%) were ‘fairly anxious’ before starting the treatment. The lowest percentage of the patients (0.69%) were ‘extremely anxious’ prior to treatment (Table 3).

**Figure 1:**
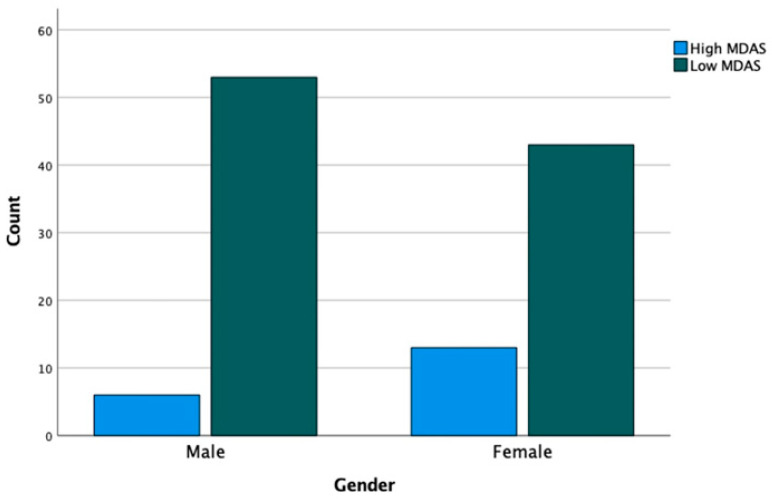
Distribution of the level of anxiety between gender.

Pre-treatment dental anxiety was compared between the gender by the Wilcoxon U test, and it showed a significant difference in all questions. Female patients were more anxious before treatment compared to male patients (Table 4). The chi-square test was used to assess the distribution of pre-treatment questionnaires between gender. It showed that there are significant differences in the responses to the MDAS questionnaire between males and females. It also showed that the majority of the female patients were extremely anxious about the tooth being drilled and local anesthesia. However, the majority of the male patients responded as ‘slightly anxious’ for all the questions (Table 5). In addition, there were a total of 19 and 96 patients were classified as having high-level anxiety and low-level anxiety, respectively. The chi-square test was also used to assess the distribution of the level of anxiety between gender which did not exhibit any significant differences. The distribution of the level of anxiety and gender is presented in Figure 1. Moreover, regression was performed to assess the influence of sociodemographic factors on dental anxiety. It showed that age and gender significantly influence the level of anxiety; however, treatment type has no effect on dental anxiety (Table 6).

After completing the treatment, a post-treatment questionnaire was provided to all patients. The frequency of their responses showed in Table 6. According to the post-treatment questionnaire, anxiety levels decreased for the majority of the patients after the treatment. A total of 44.30% of patients were anxious about the time duration of the treatment. Moreover, the presence of supervisors decreases anxiety levels for the majority of the patients (39.90%). The majority of the patients (34.80%) received endodontic treatment on their first visit with the interns (Table 7). However, none of the patients added any additional comments regarding the treatment in the open-ended question.

**Figure 2:**
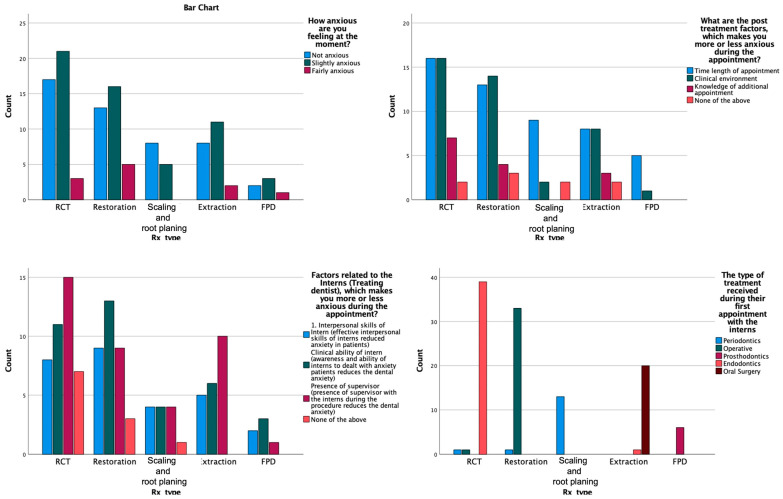
Distribution of the responses to the post-treatment questionnaire and treatment received.

The chi-square test was used to assess the distribution of post-treatment questionnaires between gender. It showed that only responses to ‘How anxious are you feeling at the moment?’ has significant influence by gender. Other responses to the post-treatment questionnaire were not significantly different between gender (Table 8). Moreover, the relation between the treatment received and the post-treatment questionnaire was assessed by the chi-square test which showed that types of treatment have no effect on the responses to the post-treatment questionnaire (Figure 2).

## 4. Discussion

The current study aimed to assess the dental anxiety who participated in the intern dental clinic at the College of Dentistry, Prince Sattam Bin Abdulaziz University. The outcome of the current study revealed that females were more anxious before starting the treatment compared to males. Age and gender have a significant association with dental anxiety; however, treatment type has no association with dental anxiety. Post-treatment questionnaire responses are not significantly different when compared to gender except one question. Moreover, the types of treatment received are also not associated with the responses to the post-treatment questionnaire.

There are many dental anxiety scales available to assess the level of dental anxiety experienced by individuals [9]. Dental anxiety scale (DAS) [44] or modified dental anxiety scale (MDAS) [14] are the most used questionnaire for dental anxiety, though these questionnaires possess some limitations. The Index of dental anxiety and fear scale (IDAF-4C) was proposed to overcome the limitations of these questionnaires which is robust; however, this scale is not extensively used in dental research [5,9]. This study used the MDAS questionnaire likewise the previous studies [25,38,39,40]. The current was conducted in the middle east where the most spoken language is Arabic and the Arabic version of MDAS is already a valid questionnaire and is used predominantly in Arabic-speaking countries [25,27,39]. However, there are many expatriates working in the middle east who cannot comprehend the Arabic language. Therefore, the current study used the original English version of the MDAS questionnaire as well as the post-treatment questionnaire. Though no expats were seeking treatment that could be treated by the interns at the time of the data collection period of this study; hence, only local people were included in this study.

The current study showed that females were more anxious before the treatment compared to males. This outcome is similar to the previous studies on dental anxiety where female patients showed higher scores for dental anxiety in the MDAS questionnaire [7,37,40,45]. Previous studies proved that females have fewer pain thresholds that increase the fear of having dental treatment [46]. This might be the reason for presenting higher MDAS scores by the females in this study. Moreover, a previous study established that females are more anxious about the administration of local anesthesia and tooth drills [47]. This statement correspondences with the outcome of the current study. In the MDAS questionnaire, it showed that a total of 21.40% and 48.20% of female patients were extremely anxious about the tooth drilled and local anesthesia, respectively. Moreover, it was also mentioned in the literature that pain is associated with the vibrating sensation and injection in dental treatment which ultimately increases the anxiousness of the patients [33,48]. However, patients are more likely to have less anxiety for even complex treatments that do not require anesthesia or tooth preparation like orthodontics [49] or bleaching procedures [50].

In this study, the patient age range was 18 to 59 years with a mean age of 35 ± 11.34 years. A previous study stated that dental anxiety has a correlation with age range. Younger people tend to feel dental anxiety than young adults [40,51,52]. Though this is not appropriate for every incidence [7]. The current study showed a significant association between age and dental anxiety; however, the current study only included adult patients. Unlike previous studies [43,50], the age range was not divided into further groups; therefore, which specific range of ages showed more dental anxiety could not determine in this study.

There were five types of treatment was provided to the patients according to their needs. The majority of the patients received root canal therapy which required drilling and oftentimes administration of local anesthesia. Moreover, the least frequent treatment provided was fixed partial denture where the use of drilling and local anesthesia is less. However, types of treatment did not show any association with MDAS. This outcome complies with the outcome of the previous study [40]. Though there were some contrasting findings available where it mentioned that complex treatment increased dental anxiety [33,53].

Though there are many previous studies were conducted on dental anxiety using the MDAS questionnaire [10,11,12], not many studies compared the pre-treatment and post-treatment anxiety levels. The current study used a post-treatment questionnaire [40] to assess the anxiety level after the treatment and which factor makes the patients more anxious. The outcome of this study showed that 48.70% of patients were slightly anxious after the treatment and none of the patients were very anxious or extremely anxious after the treatment. However, similar to the pre-treatment anxiety level, females were more anxious compared to males.

The length of the appointment and clinical environment made the patients most anxious during the treatment. The dental procedures in the hospital setup are usually like a big open area where many doctors attended to their patients, and many patients accumulate in a single room with various problems. Therefore, it is normal to feel anxious about the clinical environment especially if the patient is the first time having dental treatment. In addition, dental treatments are generally time-consuming and a longer duration of treatment time increases the anxiety level [54]. Hence, the length of appointment increased anxiety among the patients is a valid outcome as per the aforementioned study.

It was observed that the presence of a supervisor during the treatment procedure reduced the stresses during the dental treatment among patients. Moreover, the clinical ability of the interns’ interpersonal skills of interns was also reducing the anxiety level of the patients. It advocated that patients were confident about the clinical skills of the intern dental doctors. However, patients were more comforted as the supervisors were present during the treatment procedures. The previous study used the post-treatment questionnaire conducted on dental students [40]. Though the clinical skills of the intern dental doctors seem more polished than the dental students, the outcome of that study was similar to the current study. Moreover, it stated in the previous report that previous experiences with dental treatment influence the future dental experience. The more pleasant the previous dental experience was the fewer anxiety patients would feel in their future appointment [55]. Thus, due to positive outcomes for the skills and interpersonal skills of intern dental doctors, it could be assumed that patients would be less anxious about their future dental appointment.

The current study included all the samples in the convenient sampling technique until the data collection period of the study. However, appropriate sample size calculation could have scientifically strengthened the outcome of the study. Instead of using only MDAS questionnaire, using additional dental anxiety and fear scale questionnaire could have compared the outcome. Moreover, including participants from different races and compare the anxiety level among different races could also provide improved insight to the current study. The level of dental anxiety is different from younger patients and adult patients. The current study only included adult patients in this study. Including different ranges of patients could alter the outcome of the current study. Therefore, further study should have conducted to minimize the limitations of the current study. In future studies, dental anxiety could assess among multi-centers, including several Universities with larger sample size irrespective of races. Different dental anxiety questionnaires should also be assessed and compared along with the factors that might potentially impact the dental anxiety.

## 5. Conclusions

Female patients experienced more anxiety compared to male patients. Age and gender play an important role in dental anxiety; however, types of treatment are not associated with pre-treatment and post-treatment dental anxiety.

## Figures and Tables

**Table 1:** Inclusion and exclusion criteria of the patients.

Inclusion Criteria	Exclusion Criteria
Patients who needed dental treatment which could be provided by the dental intern doctors	Patients with any systemic disease
Ages between 18 to 65 years with any gender	Patients age < 18 years and >65 years
Patients who were able to write, speak and read English and Arabic	Patients who cannot read, speak and write English and Arabic
Willing to participate and gave consent to be treated by the intern dental doctors	Patients with cognitive difficulties
	Pregnant patients
	Patients who required to be attended by specialists
	Patients who were taking anxiety medicine were eliminated from the current study

**Table 2:** Descriptive statistics of the participants.

Variables		Frequency	Percentage (%)
Age (Mean ± SD)	35 ± 11.34		
Gender	Male	59	51.30
	Female	56	48.70
Treatment modalities	RCT	41	35.70
	Restoration	34	29.60
	SRP	13	11.30
	Extraction	21	18.30
	FPD	6	5.20
MDAS	High level	19	16.50
	Low level	96	83.50

SD: standard deviation; RCT: root canal therapy; SRP: surgical root planning; FDP: fixed partial denture; MDAS: modified dental anxiety scale.

**Table 3:** Frequency of the response to the pre-treatment MDAS questionnaire.

Anxiety Level	Frequency (%)					
	Q1	Q2	Q3	Q4	Q5	Total
Not Anxious	26 (22.60)	17 (14.80)	27 (23.50)	17 (14.80)	16 (13.90)	103 (17.91)
Slightly anxious	47 (40.90)	36 (31.30)	35 (30.40)	39 (33.90)	21 (18.30)	178 (30.96)
Fairly anxious	34 (29.60)	41 (35.70)	38 (33.00)	41 (35.70)	42 (35.50)	196 (34.09)
Very anxious	8 (7.00)	21 (18.30)	15 (13.00)	14 (12.20)	36 (31.30)	94 (16.35)
Extremely anxious	0 (0.00)	0 (0.00)	0 (0.00)	4 (3.50)	0 (0.00)	4 (0.69)

Q1: If you go to the dentist for treatment tomorrow, how would you feel; Q2: If you were sitting in the waiting room (waiting for treatment), how would you feel; Q3: If you were about to have a tooth drilled, how would you feel; Q4: If you were about to have your teeth scaled and polished (teeth cleaning), how would you feel; Q5: If you were to about to have the local anesthetic injection in your gum above an upper back tooth, how would you feel; %: percentage.

**Table 4:** Comparison of pre-treatment dental anxiety between gender.

MDAS	Gender	Mean Rank	Z	*p*
Q1	Male	48.44	−3.338	0.001 *
	Female	68.07		
Q2	Male	47.77	−3.531	0.0001 *
	Female	68.78		
Q3	Male	43.54	−4.974	0.0001 *
	Female	73.23		
Q4	Male	48.86	−3.160	0.002 *
	Female	67.63		
Q5	Male	45.64	−4.072	0.0001 *
	Female	71.02		
Total score	Male	42.78	−5.044	0.0001 *
	Female	74.04		

MDAS: modified dental anxiety scale; Q1: If you go to the dentist for treatment tomorrow, how would you feel; Q2: If you were sitting in the waiting room (waiting for treatment), how would you feel; Q3: If you were about to have a tooth drilled, how would you feel; Q4: If you were about to have your teeth scaled and polished (teeth cleaning), how would you feel; Q5: If you were to about to have the local anesthetic injection in your gum above an upper back tooth, how would you feel; Z: Z statistics; *p*: *p* value; *: statistical significance (<0.05).

**Table 5:** Frequency of the responses to the pre-treatment questionnaire between gender.

Questions	Responds	Male (%)	Female (%)	*p*
Q1	Not Anxious	22 (37.30)	4 (7.10)	0.002 *
	Slightly anxious	21 (35.60)	26 (46.4)	
	Fairly anxious	13 (17.40)	21 (37.50)	
	Very anxious	3 (5.10)	5 (8.90)	
	Extremely anxious	0 (0.00)	0 (0.00)	
Q2	Not Anxious	14 (23.70)	3 (5.40)	0.002 *
	Slightly anxious	23 (39.00)	13 (23.20)	
	Fairly anxious	14 (23.70)	27 (48.20)	
	Very anxious	8 (13.60)	13 (23.30)	
	Extremely anxious	0 (0.00)	0 (0.00)	
Q3	Not Anxious	0 (0.00)	0 (0.00)	0.0001 *
	Slightly anxious	23 (39.00)	4 (7.10)	
	Fairly anxious	21 (35.60)	14 (25.00)	
	Very anxious	12 (20.30)	26 (46.40)	
	Extremely anxious	3 (5.10)	12 (21.40)	
Q4	Not Anxious	11 (18.60)	6 (10.70)	0.009 *
	Slightly anxious	26 (44.10)	13 (23.20)	
	Fairly anxious	18 (30.50)	23 (41.10)	
	Very anxious	2 (3.40)	12 (21.40)	
	Extremely anxious	2 (3.40)	2 (3.60)	
Q5	Not Anxious	0 (0.00)	0 (0.00)	0.0001 *
	Slightly anxious	14 (23.70)	2 (3.60)	
	Fairly anxious	13 (22.00)	8 (14.30)	
	Very anxious	23 (39.00)	19 (33.90)	
	Extremely anxious	9 (15.30)	27 (48.20)	

Q1: If you go to the dentist for treatment tomorrow, how would you feel; Q2: If you were sitting in the waiting room (waiting for treatment), how would you feel; Q3: If you were about to have a tooth drilled, how would you feel; Q4: If you were about to have your teeth scaled and polished (teeth cleaning), how would you feel; Q5: If you were to about to have the local anesthetic injection in your gum above an upper back tooth, how would you feel; *p*: *p* value; *: statistical significance (<0.05); %: percentage.

**Table 6:** Influence of sociodemographic factors and dental anxiety.

Dental Anxiety	Variables	F	df	*p*
**MDAS**	Age	6.917	1	0.010 *
	Gender	9.289	1	0.003 *
	Treatment type	1.665	4	0.164

F: F statistics; df: degree of freedom; *p*: *p* value; *: significant differences (<0.05); MDAS: modified dental anxiety scale.

**Table 7:** Frequency of the responses to the post-treatment questionnaire.

Questions	Responses	Frequency	Percentage (%)
How anxious are you feeling at the moment?	Not Anxious	48	41.70
	Slightly anxious	56	48.70
	Fairly anxious	11	9.60
	Very anxious	0	0.00
	Extremely anxious	0	0.00
What are the post-treatment factors, which make you more or less anxious during the appointment?	Time length of appointment	51	44.30
	Clinical environment	41	35.70
	Knowledge of additional appointment	14	12.20
	None of the above	9	7.80
Factors related to the Interns (Treating dentist), which make you more or less anxious during the appointment?	Interpersonal skills of Intern (effective interpersonal skills of interns reduced anxiety in patients)	28	24.3
	The clinical ability of the intern (awareness and ability of interns to deal with anxiety patients reduce dental anxiety)	37	32.20
	Presence of supervisor (presence of supervisor with the interns during the procedure reduces dental anxiety)	39	39.90
	None of the above	11	9.60
The type of treatment received during their first appointment with the interns	Periodontics	15	13.00
	Operative	34	29.60
	Prosthodontics	6	5.20
	Endodontics	40	34.80
	Oral Surgery	20	17.40
Any other comments regarding the experience during and after treatment? Open-ended question		0	0.00

**Table 8:** Frequency of the responses to a post-treatment questionnaire between gender.

Questions	Responses	Male (%)	Female (%)	*p*
How anxious are you feeling at the moment?	Not Anxious	32 (54.20)	16 (28.60)	0.020 *
	Slightly anxious	23 (39.00)	33 (58.90)	
	Fairly anxious	4 (6.80)	7 (12.50)	
	Very anxious	0 (0.00)	0 (0.00)	
	Extremely anxious	0 (0.00)	0 (0.00)	
What are the post-treatment factors, which make you more or less anxious during the appointment?	Time length of appointment	32 (54.20)	19 (33.90)	0.061
	Clinical environment	16 (27.10)	25 (44.60)	
	Knowledge of additional appointment	5 (8.50)	9 (16.10)	
	None of the above	6 (10.20)	3 (5.40)	
Factors related to the Interns (Treating dentist), which makes you more or less anxious during the appointment?	Interpersonal skills of Intern (effective interpersonal skills of interns reduced anxiety in patients)	18 (30.50)	10 (17.90)	0.051
	The clinical ability of the intern (awareness and ability of interns to deal with anxiety patients reduce dental anxiety)	13 (22.00)	24 (42.90)	
	Presence of supervisor (presence of supervisor with the interns during the procedure reduces dental anxiety)	20 (33.90)	19 (33.90)	
	None of the above	8 (13.60)	3 (5.40)	
The type of treatment received during their first appointment with the interns	Periodontics	8 (13.60)	7 (12.50)	0.516
	Operative	17 (28.80)	17 (30.40)	
	Prosthodontics	1 (1.70)	5 (8.90)	
	Endodontics	22 (37.30)	18 (32.10)	
	Oral Surgery	11 (18.60)	9 (16.10)	

“*: Significant difference”.

## Data Availability

The data presented in this study are available on request from the corresponding author.

## Supplementary Materials

The following supporting information can be downloaded at: https://www.mdpi.com/article/10.3390/medicina59071284/s1, File S1: Post Operative Questionnaire. File S2: Preoperative questionnaire MDAS.
