# Supplementary material for: Pre-Treatment and Post-Treatment Dental Anxiety in Patients Visiting Intern Dental Clinic
Source: Medicina (Kaunas). 2023 Jul 11;59(7):1284. doi: 10.3390/medicina59071284 (PMC10384008; doi:10.3390/medicina59071284)
Supplement: Supplementary file 1 [file medicina-59-01284-s001.zip › medicina-2459395-Supplementary File S1-Post Operative Questionairre.pdf]

POST OPERATIVE QUESTIONNAIRE

1. **How anxious are you feeling at the moment?**
   1 = not anxious,
   2 = slightly anxious
   3 = fairly anxious
   4 = very anxious
   5 = extremely anxious

2. **What are the post treatment factors, which makes you more or less anxious during the appointment?**
   1. Time length of appointment,
   2. Clinical environment
   3. Knowledge of additional appointment
   4. None of the above

3. **Factors related to the Interns (Treating dentist), which makes you more or less anxious during the appointment?**
   1. Interpersonal skills of Intern (effective interpersonal skills of interns reduced anxiety in patients)
   2. Clinical ability of intern (awareness and ability of interns to dealt with anxiety patients reduces the dental anxiety)
   3. Presence of supervisor (presence of supervisor with the interns during the procedure reduces the dental anxiety)
   4. None of the above

4. **The type of treatment received during their first appointment with the interns?**
   1. Periodontics
   2. Operative
   3. Prosthodontics
   4. Endodontics
   5. Oral Surgery

5. **Any other comments regarding the experience during and after treatment? Open ended question**
