# Supplementary material for: Pre-Treatment and Post-Treatment Dental Anxiety in Patients Visiting Intern Dental Clinic
Source: Medicina (Kaunas). 2023 Jul 11;59(7):1284. doi: 10.3390/medicina59071284 (PMC10384008; doi:10.3390/medicina59071284)
Supplement: Supplementary file 1 [file medicina-59-01284-s001.zip › medicina-2459395-Supplementary File S2-Preoperative questionnaire MDAS.pdf]

Part II: Modified Dental Anxiety Scale

CAN YOU TELL US - HOW ANXIOUS YOU GET, IF AT ALL, WITH YOUR DENTAL VISIT?

Please indicate by inserting "X" in the appropriate box

1. If you went to the dentist for TREATMENT TOMORROW, how would you feel?

- ☐ Not Anxious
- ☐ Slightly Anxious
- ☐ Fairly Anxious
- ☐ Very Anxious
- ☐ Extremely Anxious

2. If you were sitting in the WAITING ROOM (waiting for treatment), how would you feel?

- ☐ Not Anxious
- ☐ Slightly Anxious
- ☐ Fairly Anxious
- ☐ Very Anxious
- ☐ Extremely Anxious

3. If you were about to have a TOOTH DRILLED, how would you feel?

- ☐ Not Anxious
- ☐ Slightly Anxious
- ☐ Fairly Anxious
- ☐ Very Anxious
- ☐ Extremely Anxious

4. If you were about to have your teeth SCALED AND POLISHED (teeth cleaning) , how would you feel?

- ☐ Not Anxious
- ☐ Slightly Anxious
- ☐ Fairly Anxious
- ☐ Very Anxious
- ☐ Extremely Anxious

5. If you were to about to have LOCAL ANAESTHETIC INJECTION in your gum, above an upper back tooth, how would you feel?

- ☐ Not Anxious
- ☐ Slightly Anxious
- ☐ Fairly Anxious
- ☐ Very Anxious
- ☐ Extremely Anxious
